# LPS Inhibits Fatty Acid Absorption in Enterocytes through TNF-α Secreted by Macrophages

**DOI:** 10.3390/cells8121626

**Published:** 2019-12-12

**Authors:** Heyuan Liu, Lixia Kai, Huahua Du, Xinxia Wang, Yizhen Wang

**Affiliations:** 1 The Key Laboratory of Molecular Animal Nutrition, Ministry of Education, Hangzhou 310058, China; 11617018@zju.edu.cn (H.L.); 21817402@zju.edu.cn (L.K.); huahuadu@zju.edu.cn (H.D.); 2 The Key Laboratory of Animal Nutrition and Feed, Ministry of Agriculture, Hangzhou 310058, China; 3 The Key Laboratory of Animal Nutrition and Feed Science of Zhejiang Province, Zhejiang University, 866 Yuhang Tang Road, Hangzhou 310058, China

**Keywords:** lipopolysaccharide, fatty acids absorption, macrophages, TNF-α

## Abstract

Diarrhea, such as steatorrhea, could result from fat absorption disorders, which could be caused by many factors, including *Escherichia coli* infection. However, it is not clear how *E. coli* affects fatty acid absorption in animals. Lipopolysaccharide (LPS), as one of the main pathogenic components of *E. coli*, is the main cause of the virulence of *E. coli*. Therefore, we used LPS to explore the underlying mechanism of *E. coli* that causes the inhibition of fatty acid absorption in the intestine. In this study, we found that LPS caused apoptosis of intestinal epithelial cells in mice. Further, caspase-3 activation caused the inhibition of fatty acid absorption in the intestinal porcine enterocyte cell line (IPEC-J2). However, direct treatment of LPS did not induce any significant change in fatty acid absorption in IPEC-J2. We then prepared conditioned medium of LPS-treated porcine macrophage cell line (3D4/2) for incubating IPEC-J2, as LPS initiates inflammation by activating immune cells. The conditioned medium decreased fatty acid absorption and caspase-3 activation in IPEC-J2. While inhibiting the activation of caspase-3 in IPEC-J2, conditioned medium no longer caused serious deficiency of fatty acid absorption. As IL-1β, IL-6, and TNF-α in conditioned medium increase significantly, IPEC-J2 was treated with IL-1β, IL-6, and TNF-α, respectively. Only TNF-α induced caspase-3 activation in IPEC-J2. Reducing the secretion of TNF-α in 3D4/2, there was no obvious activation of caspase-3 in IPEC-J2, and fatty acid absorption recovered effectively. Based on the above results, we hold the opinion that LPS does not suppress fatty acid absorption directly in the intestine, but may work on macrophages that secrete cytokines, such as TNF-α, inducing caspase-3 activation and finally leading to the inhibition of fatty acid absorption in intestine.

## 1. Introduction

Diarrhea could be induced by fatty acid (FA) malabsorption, which is one of the main reasons leading to malnutrition in children [1]. In recent research, it was found that children with celiac disease carried more *Escherichia coli* in their feces [2], and *E. coli* infection is one of the common causes inducing steatorrhea [3]. However, the mechanism by which *E. coli* induces diarrhea by blocking FA absorption is not clear.

Lipopolysaccharide (LPS) is found in the outer membrane of many (but not all) gram-negative bacteria, contributing greatly to the structural integrity of the bacteria [4]. LPS mainly comprises three parts: O antigen (or O polysaccharide), core oligosaccharide, and Lipid A [5,6]. Lipid A, anchoring LPS into the bacterial membrane, is responsible for much of the toxicity of gram-negative bacteria [6]. Therefore, LPS has often been studied in research of the mechanisms by which bacteria act on the body. LPS, as the prototypical endotoxin, is recognized by the CD14/TLR4/MD2 receptor complex, causing uncontrolled activation of mammalian immune systems with production of inflammatory mediators [7]. For instance, in macrophages, LPS promotes the secretion of pro-inflammatory cytokines, including IL-1β, IL-6, and TNF-α, inducing an inflammation reaction [8]. Once bacterial cells are lysed by the immune system, fragments of membrane containing LPS released into circulation can cause fever, diarrhea, and possibly fatal endotoxic shock (also called septic shock) [9,10]. Because of such severe inflammation, LPS gives rise to cell death, such as via apoptosis [11,12].

FAs are the main nutrients for the body and participate in various life activities. Glycerophospholipids, containing two molecules of FAs, are the main structural component of biological membranes, which can be divided into sub-regions of cells according to their function [13]. Triglycerides, containing three molecules of FAs, are the major form of energy storage, both in animals and plants [14]. Furthermore, “fat-soluble” vitamins rely on lipids to complete the process of absorption [15]. FA absorption in diet relies on enterocytes, a kind of polarized intestinal epithelia with dense microvilli [16]. Microvilli form brush borders rich in transporters for nutrient transportation, such as scavenger receptor cluster of differentiation 36 (CD36, also named fatty acid translocase) and fatty acid transporter protein 4 (FATP4) for long-chain FA (LCFA) transportation. It is considered that CD36 facilitates FA and cholesterol absorption in enterocytes [17], in addition to contributing to inflammatory responses and atherothrombotic diseases [18]. FATP4 is largely expressed in the brush border of intestinal epithelial cells and mainly participates in the transmembrane transport of dietary fatty acids [19]. Studies have shown that FATP4 can significantly promote the absorption of fatty acids [19,20]. Dietary fats are emulsified and resolved into smaller lipid molecules, mainly 2-monoacylglycerol and free fatty acids, by bile and pancreatic lipase in the intestine [21]. FAs are absorbed into enterocytes through FATP4 and CD36, resynthesized as triglycerides, and further combined into chylomicrons with apolipoproteins [22,23]. Chylomicrons are secreted into lymphatic vessels and flow into the blood through the hepatic portal vein, and triglycerides in chylomicrons are finally transferred into various tissues of the body [24]. Once excessive FAs are absorbed, chylomicronemia clearly appears in the blood [25].

In the present study, fatty acid malabsorption in the intestine was induced by LPS (5 mg/kg) in circulation. To explore the underlying mechanism, we designed the experiments in vitro. The results showed that the conditioned medium containing cytokines from LPS-treated macrophages caused apoptosis activation, followed by the decrease of CD36 and FATP4 expression, and the inhibition of fatty acid absorption in intestinal epithelial cells.

## 2. Material and Methods

### 2.1. Animals and Design of In Vivo Experiments

C57BL/6 mice and Sprague-Dawley (SD) rats were housed under 12 h light and 12 h dark for one week with food and water available ad libitum. The animal experimental protocols were approved by the Animal Care and Use Committee of Zhejiang University (Ethic Committee approval number: ZJU20160396).

Mice were injected with 100 μL sterile saline solution containing 5 mg/kg of *E. coli* LPS (Sigma–Aldrich, St. Louis, MO, USA) intraperitoneally. After one night, the mice were given 50 μg Bodipy-C16:0 (Life Technologies, CA, USA) or 200 μL olive oil, orally. The whole small intestines of Bodipy-C16:0-given mice were collected to estimate FA absorption by fluorescence intensity 3 h later. The blood was collected and the serum was separated by centrifugation at 4 °C, 2000× *g*, for 15 min. Tissue and contents of the intestines of the mice given olive oil were collected 3 h later for further experiments.

Rats were injected with 1 mL sterile saline solution containing 5 mg/kg of *E. coli* LPS (Sigma–Aldrich, St. Louis, MO, USA) intraperitoneally. After a night, the rats were given 2 mL olive oil. The blood of the rats was collected 3 h later, and the serum was separated by centrifugation at 4 °C, 2000× *g*, for 15 min.

### 2.2. Cell Culture and Design of In Vitro Experiments

IPEC-J2 and 3D4/2 were cultured in DMEM-Ham’s F12 (1:1) with 10% FBS and antibiotics (100 U/mL penicillin and 100 U/mL streptomycin sulfate). Cells were cultured at 37 °C with 5% CO_2_ in air. 3D4/2 were treated with or without 10 μg/mL of LPS (Sigma–Aldrich, St Louis, MO, USA) for 48 h to collect the supernatant as a conditioned medium (CM-LPS and CM-CON). IPEC-J2 cells were treated with CM for 12 h to analyze the FA absorption and apoptosis in vitro.

### 2.3. Intestinal Morphology Analysis

Tissues fixed with paraformaldehyde overnight were embedded in paraffin, and sections (5 µm) were stained with hematoxylin-eosin (H&E) staining. Images of paraffin section of the jejunum were obtained using a Leica DM3000 Microsystem (Leica Camera AG, Wetzlar, Germany). The villi height and crypt depth were measured using the Leica Application Suite version 3.7.0. Values of villi height or crypt depth were the average of 3 measurements for each mouse.

Histopathological lesions of jejunum were scored according to the previous image analysis method [26]; a pathologist and 5 scientists with basic histological experience scored the severity and extent of inflammation caused by LPS as judges.

### 2.4. Gut Permeability Assessment

Diamine oxidase (DAO) is an endoenzyme in enterocytes, and D-lactic acid is a bacterial metabolite in the intestine. As intestinal permeability increases, serum levels of DAO and D-lactic acid clearly rise. Hence, DAO and D-lactic acid can often be regarded as markers for gut permeability [27,28]. Serum concentration of DAO and D-lactic acid were measured using commercial mouse ELISA kits (Fankewei, Shanghai, P.R. China) according to the manufacturer’s instructions.

FITC-dextran 4 (FD4) (Sigma–Aldrich, Shanghai, P.R. China) was administered at 20 mL/kg body weight by oral gavage after LPS or saline administration overnight. One hour later, the mice serum was separated, and the serum fluorescence was measured by a Molecular Devices SpectraMax M5 plate reader (excitation 485 nm, emission 535 nm, San Jose, CA, USA). The serial dilution method was applied to dilute 40 mg/mL of FD4 with physiological saline in order to draw the standard curve, and the serum FD4 concentration was calculated according to the standard curve.

### 2.5. Immunohistochemistry

Tissues fixed with paraformaldehyde overnight were embedded in paraffin and sliced in sections of 5 µm thickness. Sections were dewaxed in xylole and rehydrated in graded alcohol in preparation for immunohistochemistry (IHC). IHC was performed as per the previous study [29]. The primary antibody of M1 macrophage marker, anti-CD11c (Servicebio, Wuhan, China), was used. Images of the paraffin section of the jejunum were obtained using a Leica DM3000 Microsystem (Leica Camera AG, Wetzlar, Germany).

### 2.6. Apoptosis and Pro-Inflammatory Cytokine Treatment on IPEC-J2 Cells

Caspase-3 is the executor of apoptosis in the caspase-dependent pathway [30]. PAC-1 (APExBIO, Houston, USA), a caspase-3 activator, was added to IPEC-J2 cell culture for 12 h. Z-DEVD-FMK (APExBIO, Houston, TX, USA), a caspase-3 inhibitor, was added to IPEC-J2 cell culture 2 h before the cells were treated with LPS. IL-1β, IL-6, and TNF-α in the culture supernatant of 3D4/2 were measured using commercial porcine ELISA kits according to the manufacturer’s instructions. Recombinant protein of IL-1β, IL-6, and TNF-α (all from R&D systems, Minneapolis, USA) was added to the IPEC-J2 cells culture for 24 h. Lenalidomide hydrochloride (LH) (APExBIO, Houston, TX, USA), a TNF-α inhibitor, was added to 3D4/2 cell culture, and the mRNA expression of TNF-α in 3D4/2 was then analyzed to optimize treatment concentration. The collected supernatant of 3D4/2 culture were treated with incubated IPEC-J2 cells, and FA absorption and apoptosis of IPEC-J2 were then analyzed.

### 2.7. Western Blot Analysis

The separated tissues of jejunum in mice were homogenized using a Whole Protein Extraction Kit (KeyGen Biotech, Nanjing, P.R. China), and protein concentrations were quantified using a BCA Protein Quantification Kit (KeyGen Biotech, Nanjing, P.R. China). The total protein of IPEC-J2 cells was prepared using Protein Loading Dye (Sangon Biotech, Shanghai, P.R. China).

Equivalent proteins were separated using 15% SDS-PAGE, electro-blotted onto polyvinylidene fluoride membranes, and then blocked with 5% fat-free milk. Then, membranes were incubated overnight at 4 °C with primary antibodies for CD36, FATP4, caspase-3, and β-actin (all purchased from Proteintech Group, Wuhan, P.R. China), and subsequently incubated with goat-anti-rabbit IgG secondary horseradish peroxidase-conjugated antibody (CST, Boston, MA, USA) for 1 h at room temperature. The protein blots were photographed using a Tanon 4200SF Chemiluminescent Imaging System (CliNX, Shanghai, P.R. China). Intensity of blots was quantified using ImageJ software.

### 2.8. RNA Isolation and qRT-PCR

Total RNA isolation and cDNA synthesis by reverse transcription were performed using the TRIzol reagent and a reverse transcriptase kit (Thermo Fisher Scientific, Boston, MA, USA). The mRNA levels of individual genes were determined using a SYBR PCR Master Mix (Roche, Basel, Switzerland) in the ABI StepOne Plus™ Real-time PCR System (Applied Biosystems, Foster City, CA, USA). Data was analyzed by the 2−ΔΔCt method and normalized based on GAPDH as the reference gene. The primers used in this experiment are listed in Table 1.

### 2.9. Fluorescence Microscopy

In this study, a bodipy-labeled fluorescent, Bodipy-C16:0 (D3822, Invitrogen, Shanghai, P.R. China), was used to measure the absorption of LCFA in IPEC-J2. The cells cultivated on confocal dishes were incubated in 1.5 mL working solution containing 5 μmol/L Bodipy-C16:0 for 10 min. Then, the cells were fixed in 4% paraformaldehyde solution for 15 min. After washing with saline, the cells were incubated in a ready-to-use DAPI (4 μg/L) reagent (Servicebio, Wuhan, P.R. China) for 10 min in the dark and examined with a Zeiss LSM 780 confocal microscope (Zeiss, Jena, Germany) or a IX71 inversion fluorescence microscope (Olympus, Tokyo, Japan) with excitation of 485 nm and emission of 528 nm. The pictures were analyzed using ZEN 2012 software (Zeiss, Jena, Germany).

### 2.10. Statistical Analysis

The Student’s *t*-test and one-way analysis of variance (ANOVA, post-hoc Tukey’s tests) were conducted using GraphPad Prism version 5.01 (GraphPad Software, Inc., San Diego, CA, USA) to compare the two means and the three means of the measured variables, respectively. Data are presented as mean ± SD. Statistically significant results were determined at the 0.05 confidence level (*p* < 0.05).

## 3. Results

### 3.1. LPS Leading to FA Absorption Inhibition In Vivo

**Figure 1:**
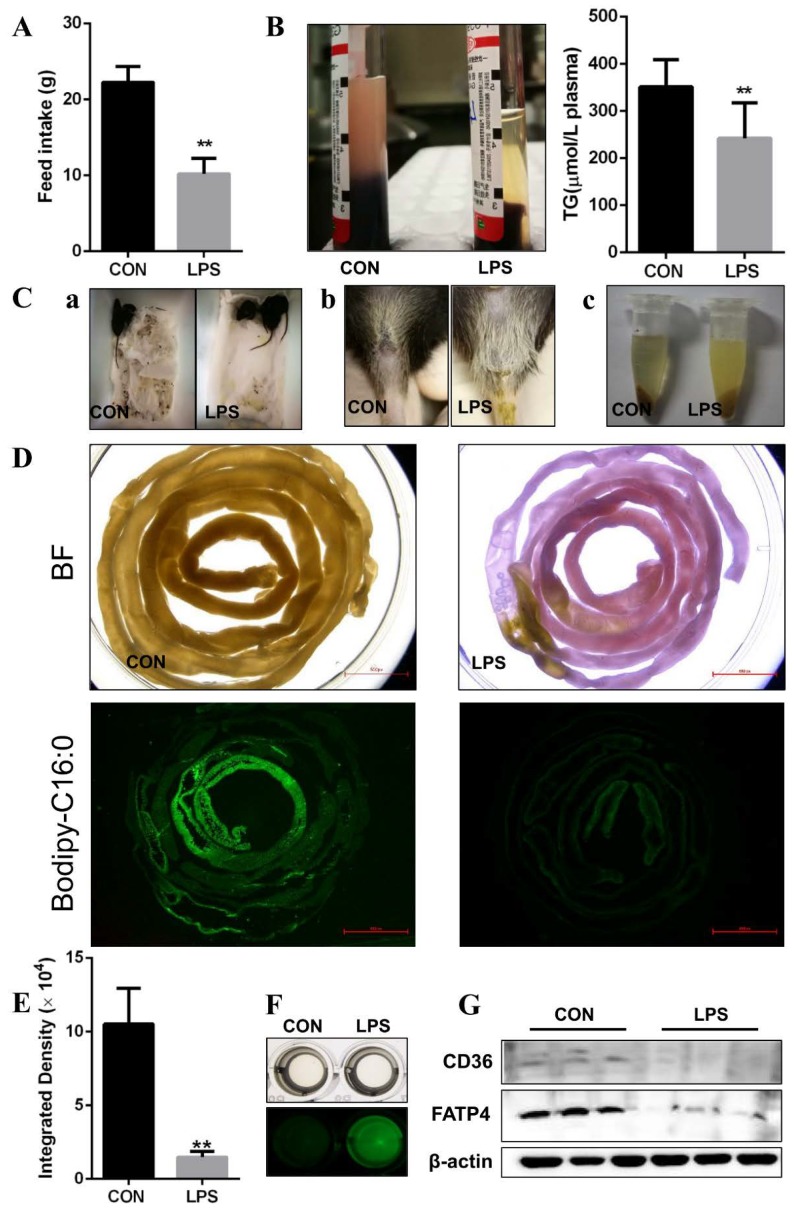
Lipopolysaccharide (LPS) induces fatty acid (FA) uptake inhibition in vivo. The group of CON and LPS: rats and mice were treated with or without LPS. (**A**) Feed intake in rats after LPS i.p. injection 24 h later (n = 4). (**B**) The apparent (left) and the triglycerides concentration (right) of serum in rats given olive oil (2 mL) orally (n = 3). (**C**) The physiological status (a), fecal matter (b), and supernatant of intestinal contents (c) of mice. (**D**) The entire small intestine of mice under white light (upper) and localization of Bodipy-C16:0 in the entire small intestine under blue light (lower) (Scale bar: 500px). (**E**) Integrated density of Bodipy-C16:0 in the entire small intestine (n = 3). (**F**) CD36 and FATP4 protein level in the intestine tissues were analyzed by western blotting (n = 3). Results were normalized with β-actin level. (**G**) Fluorescence of Bodipy-C16:0 in supernatant of intestinal contents in mice. Data were shown as mean ± SD, ** *p* < 0.01.

We first noted that LPS induced a reduction in the feed intake of rats (Figure 1A), and mental sluggishness and diarrhea in mice (Figure 1C). After the LPS-treated rats and mice were given olive oil orally, the plasma of rats did not show chylomicronemia and contained lower triglyceride (Figure 1B). The intestinal contents of mice seemed to contain more unabsorbed FAs in the cloudy supernatants (Figure 1C). To confirm the effect of LPS on FA absorption in vivo, the fluorescence intensity of the whole intestine was detected after mice were given bodipy-labeled FAs orally. The lower integrated density appeared in the intestines with congestion and swelling (Figure 1D,E), but more fluorescence was detectable in the intestinal contents (Figure 1F) after LPS administration given to mice overnight. Further, we found that the main proteins involved in transportation of FAs in the intestine, CD36 and FATP4, also decreased overall in response to LPS (Figure 1G). Taken together, these results indicate that LPS inhibits FA absorption in vivo.

### 3.2. Inhibition of FA Absorption Caused by Apoptosis

**Figure 2:**
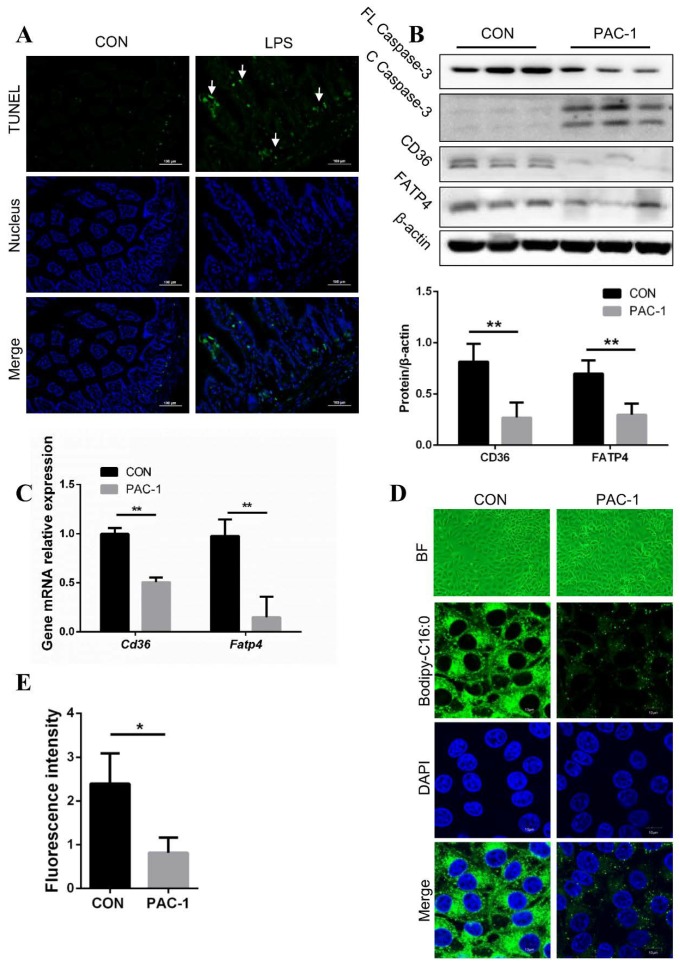
Apoptosis induces FA uptake decrease in IPEC-J2. (**A**) TUNEL staining of jejunum tissue slices in mice (n = 3, magnification: 200×). The white arrows mark the point of apoptotic signal. (**B**) Western blot analysis of full-length caspase-3 (FL Caspase-3), cleaved caspase-3 (C Caspase-3), CD36 and FATP4 expression in IPEC-J2 treated with or without PAC-1. Results were normalized with β-actin level (n = 3). (**C**) qPCR analysis of CD36 and FATP4 expression in IPEC-J2 with or without PAC-1. Results were normalized with *Gapdh* level and expressed as fold of control (n = 3). (**D**,**E**) Images of IPEC-J2 with or without PAC-1 treatment under green optical filter and FA uptake was evaluated by intracellular fluorescence intensity of BODIPY C16 by fluorescence microscope (n = 3). Data are shown as mean ± SD, * *p* < 0.05 and ** *p* < 0.01.

It has been well established in many studies that LPS induces apoptosis by activating the caspase-3 signal pathway in various cells [31]. Thus, we hypothesized that LPS suppressed FA absorption in vivo, caused by the activation of the apoptosis signal in enterocytes. As expected, the apoptosis signal was detectable in jejunal epithelial cells in LPS-treated mice using TUNEL staining (Figure 2A). To investigate whether apoptosis affected FA absorption in enterocytes, we next conducted apoptosis activation using PAC-1, an activator of caspase-3, that exhibited more cleaved caspase-3 in IPEC-J2 (Figure 2B). We then found that the mRNA and protein levels of CD36 and FATP4 decreased under apoptosis activation (Figure 2B,C). Furthermore, the fluorescence intensity of Bodipy-C16:0 representing FA absorption showed a significant decrease in PAC-1-treated IPEC-J2 (Figure 2D,E). Overall, this suggests that FA absorption inhibition may be caused by apoptosis in enterocytes.

### 3.3. No Effect on Fatty Acid Absorption in IPEC-J2 after LPS Treatment

**Figure 3:**
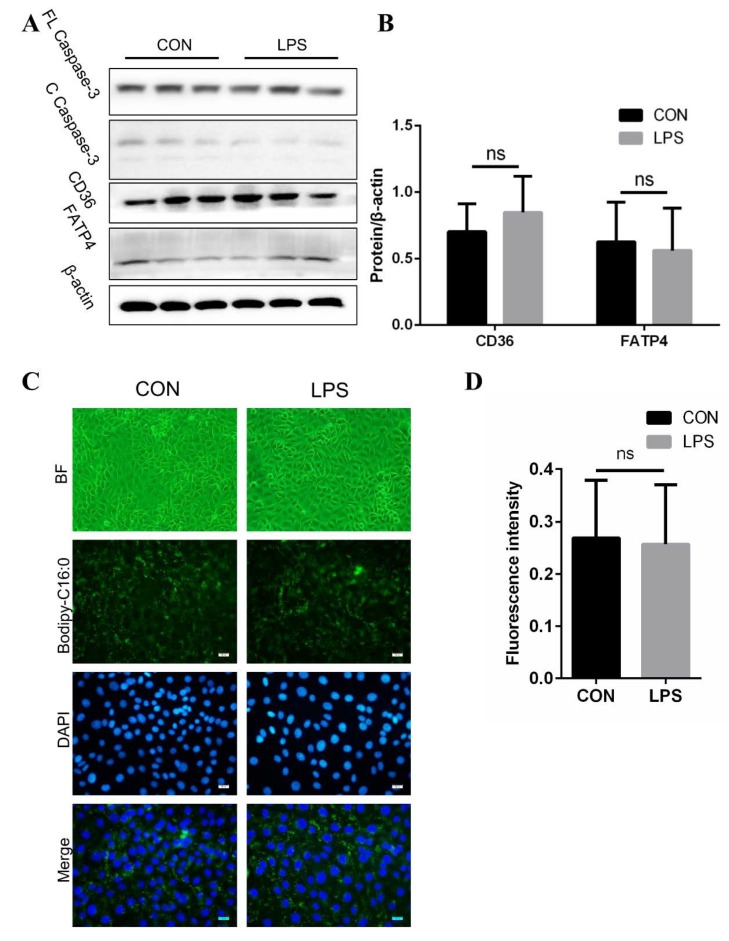
LPS has no direct effect on FA uptake in IPEC-J2. (**A**,**B**) Western blot analysis of FL Caspase-3, C Caspase-3, CD36, and FATP4 expression in IPEC-J2 treated with or without LPS. Results were normalized with β-actin level (n = 3). (**C**) Images of IPEC-J2 with or without LPS treatment under green optical filter (magnification: 20×) or blue-fluorescence (scale bar: 20 μm). (**D**) Fluorescence intensity of Bodipy-C16:0 in IPEC-J2 with or without LPS treatment (n = 3). Data are shown as mean ± SD, ns means *p* > 0.05.

To explore whether LPS inhibits FA absorption by activating apoptosis directly in IPEC-J2, we detected cleaved caspase-3 after LPS was added to IPEC-J2, while there was no obvious difference in the levels of cleaved caspase-3 between LPS-treated IPEC-J2 and control cells (Figure 3A). Furthermore, LPS treatment did not affect the protein expression of CD36 and FATP4 (Figure 3 A,B), and the fluorescence intensity of Bodipy-C16:0 appeared similarly between control cells and LPS-treated IPEC-J2, which also showed a similar morphology (Figure 3C,D). Hence, we consider that LPS did not inhibit FA absorption directly in IPEC-J2.

### 3.4. Injury of Jejunal Epithelium Caused by LPS Intraperitoneal Injection in Mice

**Figure 4:**
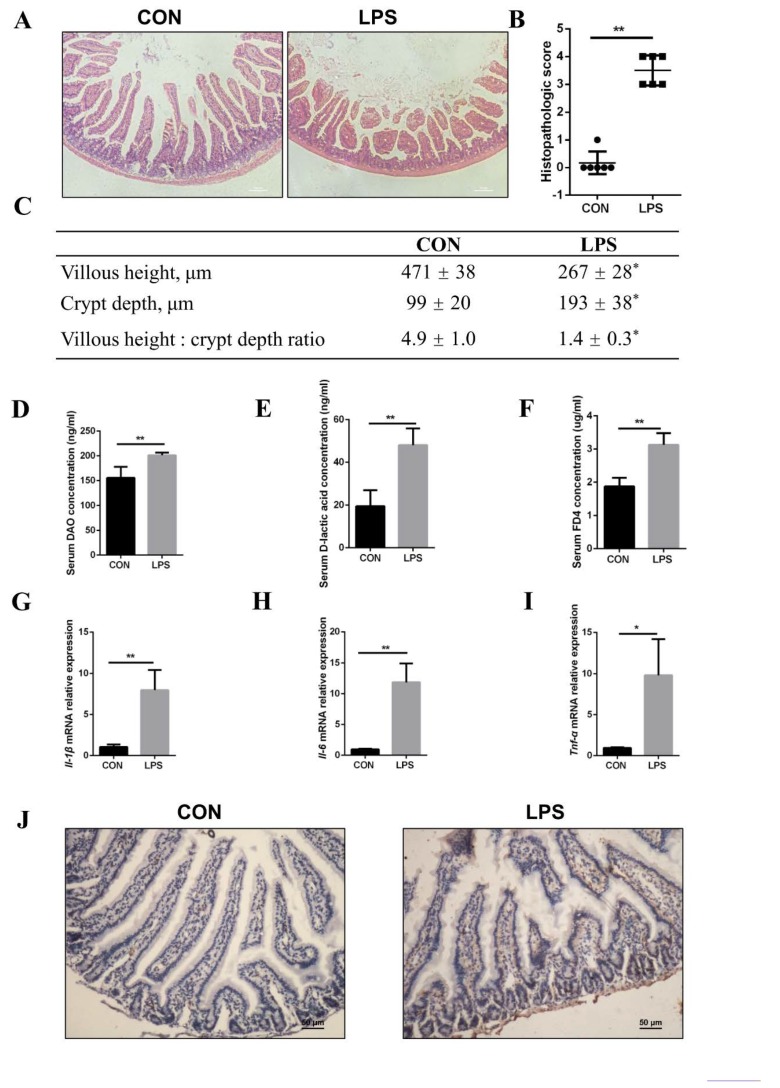
LPS injuries to intestinal morphology of jejunum. (**A**) H&E staining of the jejunum in mice treated with or without LPS (scale bar: 100 μm). (**B**) Histology of jejunums was scored based on histopathologic grading (n = 6). (**C**) The villous height and crypt depth of the jejunum were measured, and the ratio of villous height vs crypt depth was also analyzed (n = 6). (**D**–**F**) the DAO, D-lactic acid, and FD4 concentrations in serum of mice were measured (n = 6). (**G**–**I**) the mRNA expression of Il-1β, Il-6, and Tnf-α was analyzed by RT-qPCR in mice (n = 6). Results were normalized with *Gapdh* level and expressed as fold of control. (**J**) Immunohistochemical staining of M1 macrophages in jejunum with an antibody against CD11c (scale bar: 50 μm). Data are shown as mean ± SD, * *p* < 0.05 and ** *p* < 0.01.

It has been widely proved that LPS damages tissues by inducing inflammation [32,33]. Consistent with previous studies, LPS destroyed intestinal morphology in vivo. Small intestinal tissue sections from LPS-treated mice exhibited atrophic and shed villi (Figure 4A), along with serious damage of intestinal tissue according to histopathologic scores (Figure 4B). The length of intestinal villi and crypt were measured to analyze the capacity of nutrient absorption in vivo [34]. It was found that the ratio of villi height to crypt depth deceased because of shorter villi and deeper crypts in LPS-injected mice (Figure 4C). To test the effect of LPS on intestinal physical barrier integrity and inflammation response in mice, indexes reflecting intestinal permeability and inflammation were measured. The level of serum DAO, D-lactic acid, and FD4 appeared to be much higher (Figure 4D–F), and the mRNA level of pro-inflammatory factors, IL-1β, IL-6, and TNF-α also increased in LPS-treated mice (Figure 4G–I). M1 macrophages in jejunum were detected because of their abilities of producing various inflammatory factors [35]. Immunohistochemistry results showed that there were more M1 macrophages in jejunum of LPS-treated mice (Figure 4J). From the above results, it is evident that LPS has access to intestinal tissue damage, which may be mediated by inflammation.

### 3.5. Apoptosis and Fatty Acid Absorption Inhibition in IPEC-J2 Induced by CM-LPS

As LPS stimulates macrophage secreting cytokines, mainly IL-1β, IL-6, and TNF-α, to initiate inflammatory response, we next explored whether macrophages mediated the progress in which LPS induced apoptosis and FA absorption inhibition in vitro. First, 3D4/2 were treated with or without LPS to collect the cell culture as conditioned medium (CM-LPS and CM-CON). Then, the conditioned medium was used to treat IPEC-J2 to analyze apoptosis and FA absorption.

**Figure 5:**
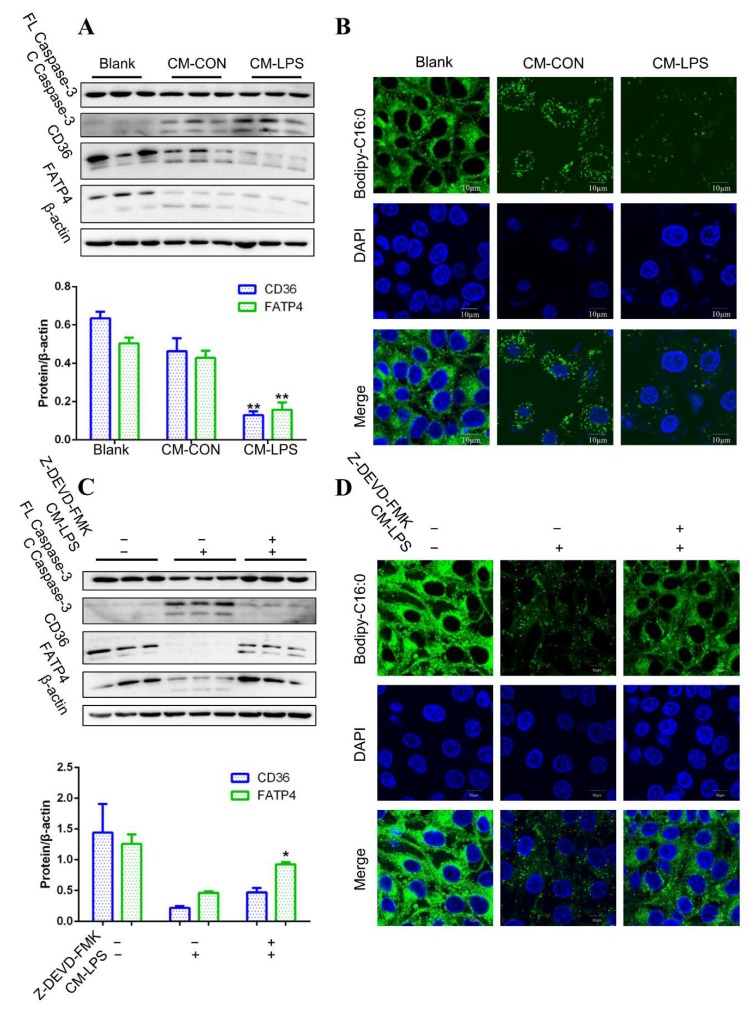
CM-LPS of PAMs induces apoptosis and FA uptake inhibition in IPEC-J2. **3D4/**2 were treated with or without 10 μg/mL of LPS (Sigma–Aldrich, St Louis, MO, USA) for 48 h to collect the supernatant as a conditioned medium (CM-LPS and CM-CON), and then the conditioned medium were used to incubate IPEC-J2. (**A**) Western blot analysis of FL Caspase-3, C Caspase-3, CD36, and FATP4 expression in IPEC-J2 treated with CM-CON, CM-LPS, or nothing. Results were normalized with β-actin level (n = 3). (**B**) Fluorescence intensity of Bodipy-C16:0 in IPEC-J2 treated with CM-CON, CM-LPS, or noting was captured by laser scanning confocal microscope technology (scale bar: 10 μm). (**C**) Western blot analysis of FL Caspase-3, C Caspase-3, CD36, and FATP4 expression in IPEC-J2 treated with CM-LPS under Z-DEVD-FMK presence or absence or nothing. Results were normalized with β-actin level (n = 3). (**D**) Fluorescence intensity of Bodipy-C16:0 in IPEC-J2 treated with CM-LPS under Z-DEVD-FMK presence or absence or nothing was captured by laser scanning confocal microscope technology (scale bar: 10 μm). Data are shown as mean ± SD, * *p* < 0.05 and ** *p* < 0.01 compared with CM-CON.

Caspase-3 was clearly cleaved after treatment with CM-LPS in IPEC-J2 (Figure 5A). CM-LPS treatment also led to lower protein expression of CD36 and FATP4 in IPEC-J2 compared with CM-CON (Figure 5A). IPEC-J2 treated with CM-LPS showed lower fluorescence intensity of Bodipy-C16:0 than that of CM-CON (Figure 5B). Together, these data suggest that CM-LPS leads to apoptosis and FA absorption inhibition in IPEC-J2. To validate whether CM-LPS induces FA absorption inhibition due to activating caspase-3, an inhibitor of caspase-3, Z-DEVD-FMK, was used to treat IPEC-J2. Cleaved caspase-3 did not appear significantly in the presence of Z-DEVD-FMK after CM-LPS treatment in IPEC-J2 (Figure 5C). Furthermore, the protein expression of CD36 and FATP4 under Z-DEVD-FMK treatment also partly recovered compared with only CM-LPS treatment (Figure 5C). Z-DEVD-FMK also ameliorated the reduction of fluorescence intensity of Bodipy-C16:0 caused by CM-LPS (Figure 5D). Taken together, these results indicate that LPS inhibited FA absorption mediated by CM-LPS activating caspase-3 in IPEC-J2.

### 3.6. TNF-α Present in CM-LPS Inducing Apoptosis of IPEC-J2

**Figure 6:**
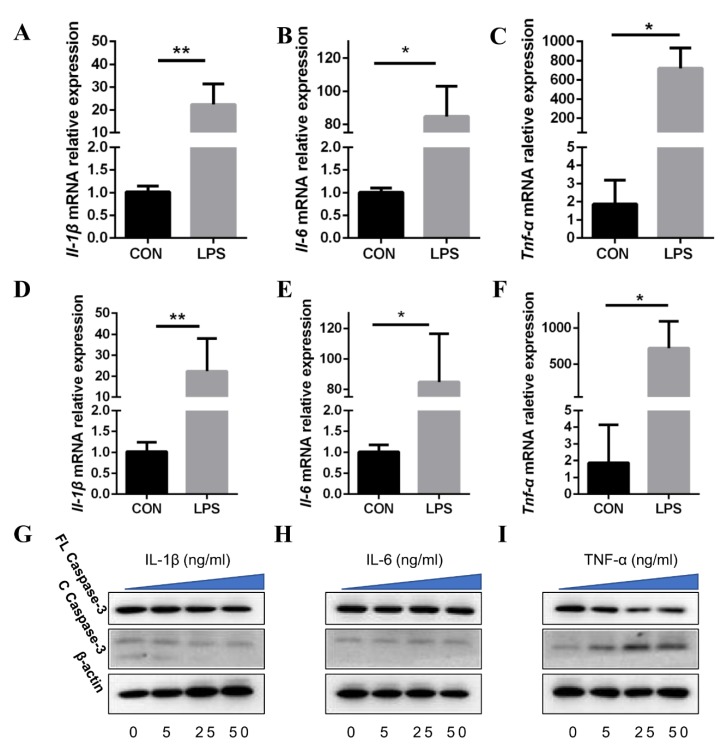
TNF-α induces caspase-3 activation in IPEC-J2. (**A**–**C**) The mRNA expression of Il-1β, Il-6 and Tnf-α was analyzed by RT-qPCR in PAMs treated with LPS (n = 3). Results were normalized with *Gapdh* level and expressed as fold of control. (**D**–**F**) The concentrations of IL-1β, IL-6, and TNF-α in culture supernatant of LPS-treated PAMs were measured by ELISA kits (n = 3). (**G**–**I**) The three cytokines were administrated on IPEC-J2 with 0, 5, 25, and 50 ng/mL directly. Caspase-3 activation in IPEC-J2 was detected by western blotting. Data are shown as mean ± SD, * *p* < 0.05 and ** *p* < 0.01.

To further explore the underlying mechanism by which LPS induces apoptosis in IPEC-J2, the cytokines, IL-1β, IL-6, and TNF-α were detected in 3D4/2. Consistent with previous studies [36], the gene expression of Il-1β, Il-6, and TNF-α in 3D4/2 increased after LPS treatment for 6 h (Figure 6A–C). Then, IL-1β, IL-6, and TNF-α in CM were measured by ELISA. Each of these three cytokines were more evident in CM-LPS than in CM-CON (Figure 6D–F). To confirm whether caspase-3 activation in IPEC-J2 was induced by the three cytokines, IL-1β, IL-6, and TNF-α were used to treat IPEC-J2, respectively. Of the three, only TNF-α aggravated cleaved caspase-3 production in IPEC-J2, which showed a tendency of positive correlation with TNF-α concentration (Figure 6G). These results demonstrate that TNF-α in CM-LPS induces caspase-3 activation in IPEC-J2.

### 3.7. Preventing the Secretion of TNF-α Leading to Better Fatty Acid Absorption

**Figure 7:**
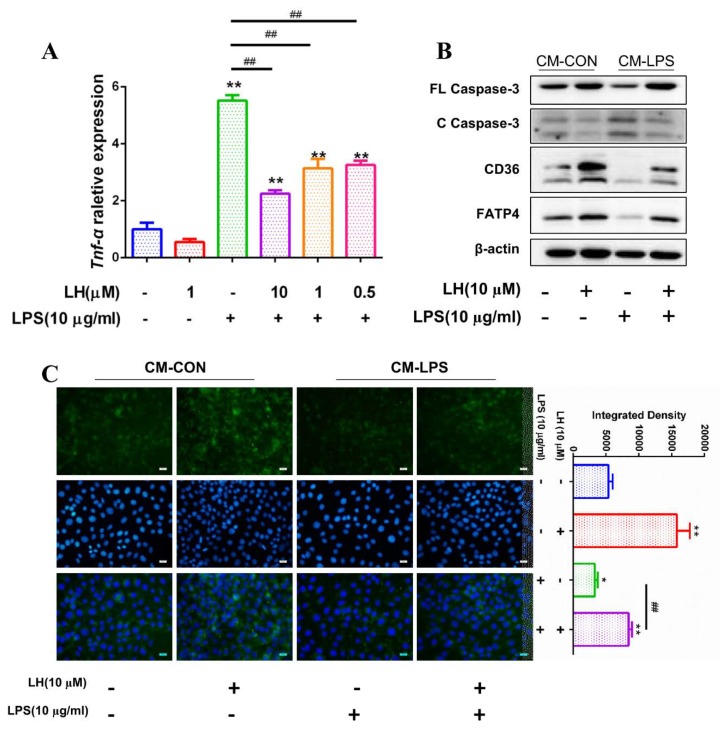
TNF-α induces FAs uptake inhibition in IPEC-J2. Lenalidomide hydrochloride (LH), an inhibitor for TNF-α, was administrated on PAMs to prevent TNF-α secretion. (**A**) The mRNA expression of *Tnf-α* was analyzed by RT-qPCR in LPS-treated PAMs with 0.5, 1, and 10 μM at the same time (n = 3). PAMs treated with nothing as blank, PAMs treated with LPS (10 μg/mL) or LH (1 μM) as control. Results were normalized with *Gapdh* level and expressed as fold of blank. (**B**) Western blot analysis of FL Caspase-3, C Caspase-3, CD36, and FATP4 expression in IPEC-J2 treated with CM-CON or CM-LPS under LH presence or absence. Results were normalized with β-actin level. (**C**) Fluorescence intensity of Bodipy-C16:0 in IPEC-J2 was captured by laser scanning confocal microscope technology (scale bar: 10 μm). Data are shown as mean ± SD, * *p* < 0.05, ** *p* < 0.01 compared with blank, ^##^ *p* < 0.01 compared with LPS control.

To ascertain whether FA absorption inhibition in IPEC-J2 is caused by TNF-α in CM-LPS, the lenalidomide hydrochloride (LH), an inhibitor of TNF-α, and LPS, were used together to incubate 3D4/2. When 10 µM LH was added, the mRNA level of Tnf-α in 3D4/2 showed the most obvious decrease compared with 0.5 µM and 1 µM LH (Figure 7A). Hence, CM-CON and CM-LPS with or without 10 µM LH were used to treat IPEC-J2, respectively. IPEC-J2 with CM-LPS and 10 µM LH administration exhibited less cleaved caspase-3 than that only treated with CM-LPS (Figure 7B). Then, the indexes representing FA absorption were measured. As expected, the protein expression of CD36 and FATP4 recovered partly (Figure 7B), and IPEC-J2 showed higher fluorescence intensity than that of only CM-LPS treatment (Figure 7C). Taken together, these results indicate that TNF-α in CM-LPS induces FA absorption inhibition in IPEC-J2.

## 4. Discussion

In this paper, we show the underlying mechanism by which LPS inhibits FA absorption in vivo. First, we noted that LPS caused apoptosis of enterocytes with TUNEL staining, consistent with the results of others. For example, LPS was used to induce apoptosis to analyze the features of LPS receptor subunits [37], and to verify *Clostridium tyrobutyricum* protected intestinal barrier function from LPS-induced apoptosis in IPEC-J2 cells [38]. The zymogen feature of caspase-3 is necessary because, if unregulated, caspase-3 activity would injure cells indiscriminately [39]. As an executioner caspase, the caspase-3 zymogen has virtually no activity until it is cleaved by an initiator caspase after apoptotic signaling events have occurred [40]. Caspase-3 has been found to be necessary for its typical role in apoptosis, where it was responsible for chromatin condensation and DNA fragmentation [41]. We found that PAC-1 activated caspase-3 in IPEC-J2, inducing FA absorption inhibition and a decrease in the protein expression of CD36 and FATP4. For caspase-3 related to DNA fragmentation, a possible mechanism may be that caspase-3 activation inhibited FA absorption through cleaving the genes of CD36 and FATP4.

LPS did not show any effect on FA absorption and CD36 and FATP4 expression, in vitro after LPS was added to the cell culture of IPEC-J2. Ghoshal et al. treated Caco-2 cells with 0.1 mg/mL unlabeled LPS overnight to assess the ability of LPS absorption [42]. In that study, such a high dosage of LPS also did not affect the function of Caco-2 cells, which is similar to the results of our study. Based on this pattern, we suspect that maybe LPS inhibited FA absorption indirectly.

LPS could be recognized by the TLR4 of immune cells, such as macrophages, to activate inflammatory signaling pathways and release pro-inflammatory factors, including IL-1β, IL-6, and TNF-α [43]. TNFR1, expressed in most tissues, is involved in death signaling as a death-domain-containing member of the TNFR superfamily [44]. TNFR1 captures TNF receptor-associated death domain (TRADD) in cytoplasm, and TRADD binds the Fas-associated protein with death domain (FADD) to recruit the cysteine protease caspase-8 [45]. A high concentration of caspase-8 induces its autoproteolytic activation and subsequent cleaving of effector caspases, such as caspase-3, finally leading to cell apoptosis [46]. An intestinal inflammatory injury mouse model contains a large number of M1 macrophages that can secrete pro-inflammatory factors [47], which is consistent with our experimental results. We noticed that the CM from the 3D4/2 with LPS stimulation, containing amounts of TNF-α, inhibited FA absorption in IPEC-J2. Moreover, IPEC-J2 cells partly recovered the function of FA absorption after the progress of TNF-α secretion was suppressed. These results conform to the previous research on TNF-α inducing apoptosis [48].

Due to the limited situation, no further study has been conducted on the reason for the reduction of CD36 and FATP4 expression caused by caspase-3. This might be due to the fragmentation of genes caused by activation of caspase-3, as noted above. In addition, the molecular weight of integrated CD36 is about 88 kD, but it was noticed that some WB revealed with anti-CD36 appear in two bands in the above results. Kim et al. found that CD36 could be modified into smaller molecules by ubiquitin [49], which may be the reason leading to the WB appearing in two bands. Finally, we foun that 3D4/2, without LPS stimulation, secreted a small amount of TNF-α. Other studies also report that macrophages can secrete limited cytokines without any treatment, such as in the research of Dlugosz et al. [50] and Sharma et al. [51]. It is possible that macrophages that secreted cytokines that caused CM in the control group inhibited FA absorption in IPEC-J2.

As one of three main nutrients, FAs participate in many physiological processes, such as storing energy, making up biological membranes, promoting nervous system development, etc. Lack of polyunsaturated FAs is associated with metabolic diseases early in life [52].

Inflammatory reaction is necessary to drive away pathogens during infections; a number of pro-inflammatory factors, including IL-1β, IL-6, and TNF-α, are secreted by macrophages. Such pro-inflammatory factors could mediate tissue damage; in particular, TNF-α can directly recognize death domain to active caspase-8/3 pathways, leading to apoptosis [45,46]. Prolonged exposure to low concentrations of TNF-α can result in cachexia, a wasting syndrome, which can be found in cancer patients [53].

In bodies of infants, which are not yet well developed, inflammation may result in malnutrition, potentially leading irreversible consequences without timely control. It is necessary to emphasize the importance of controlling inflammatory processes at the right level and not only destroying pathogens in the case of non-reversible damage caused by nutrient malabsorption.

## 5. Conclusions

The aim of this study was to investigate the potential mechanism of LPS inhibiting FA absorption in intestinal epithelium in vivo. LPS had no significant effect on fatty acid absorption when directly acting on intestinal epithelial cells. According to the results, it appears that LPS inhibits FA absorption in vivo due to the overexpressed TNF-α in macrophages through LPS stimulation. In particular, LPS induces 3D4/2 secreting TNF-α, and TNF-α then activates caspase-3 in enterocytes, leading to the decrease of protein expression of CD36 and FATP4, and the subsequent inhibition of FA absorption.

## Figures and Tables

**Table 1:** Sequences of the primers for amplifying target genes.

Gene	Forward Primer (5′-3′)	Forward Primer (5′-3′)
m ^*^-*Il-1β*	TCCAGGATGAGGACATGAGCAC	GAACGTCACACACCAGCAGGTTA
m-*Il-6*	CCACTTCACAAGTCGGAGGCTTA	CCAGTTTGGTAGCATCCATCATTTC
m-*Tnf-α*	TATGGCCCAGACCCTCACA	GGAGTAGACAAGGTACAACCCATC
m-*Gapdh*	TGTGTCCGTCGTGGATCTGA	TTGCTGTTGAAGTCGCAGGAG
p ^#^-*Il-1β*	GAGCTGAAGGCTCTCCACCTC	ATCGCTGTCATCTCCTTGCAC
p-*Il-6*	TTCACCTCTCCGGACAAAAC	TCTGCCAGTACCTCCTTGCT
p-*Tnf-α*	TTCCAGCTGGCCCCTTGAGC	GAGGGCATTGGCATACCCAC
p-*Cd36*	CCATACCCTATTCCTACCAC	AGGCTGCATCTGTACCATTA
p-*Fatp4*	TATGGTGTGGAGGTGCCAGGAA	CCGCAGGTCTGTCTTCTGTAGC
p-*Gapdh*	CAAGGAGTAAGAGCCCCTGG	GGTACATGACGAGGCAGGTC

* m means mice, # p means pig.

## References

[1] Wang T.Y., Liu M., Portincasa P., Wang D.Q. (2013). New insights into the molecular mechanism of intestinal fatty acid absorption. Eur. J. Clin. Investig..

[2] Olivares M., Benitez-Paez A., de Palma G., Capilla A., Nova E., Castillejo G., Varea V., Marcos A., Garrote J.A., Polanco I. (2018). Increased prevalence of pathogenic bacteria in the gut microbiota of infants at risk of developing celiac disease: The PROFICEL study. Gut Microbes.

[3] Cheifetz A.S. (2011). Oxford American Handbook of Gastroenterology and Hepatology.

[4] Sutcliffe I.C. (2010). A phylum level perspective on bacterial cell envelope architecture. Trends Microbiol..

[5] Wang X., Ribeiro A.A., Guan Z., McGrath S.C., Cotter R.J., Raetz C.R. (2006). Structure and biosynthesis of free lipid A molecules that replace lipopolysaccharide in Francisella tularensis subsp. novicida. Biochemistry.

[6] Anwar M.A., Choi S. (2014). Gram-negative marine bacteria: Structural features of lipopolysaccharides and their relevance for economically important diseases. Mar. Drugs.

[7] Kilar A., Dornyei A., Kocsis B. (2013). Structural characterization of bacterial lipopolysaccharides with mass spectrometry and on- and off-line separation techniques. Mass Spectrom. Rev..

[8] Lichtman A.H., Abbas A.K. (2006). Basic Immunology: Functions and Disorders of the Immune System.

[9] Opal S.M. (2010). Endotoxins and other sepsis triggers. Contrib. Nephrol..

[10] Beutler B., Rietschel E.T. (2003). Innate immune sensing and its roots: The story of endotoxin. Nat. Rev. Immunol..

[11] Mandal P., Feng Y., Lyons J.D., Berger S.B., Otani S., DeLaney A., Tharp G.K., Maner-Smith K., Burd E.M., Schaeffer M. (2018). Caspase-8 Collaborates with Caspase-11 to Drive Tissue Damage and Execution of Endotoxic Shock. Immunity.

[12] Tucureanu M.M., Rebleanu D., Constantinescu C.A., Deleanu M., Voicu G., Butoi E., Calin M., Manduteanu I. (2018). Lipopolysaccharide-induced inflammation in monocytes/macrophages is blocked by liposomal delivery of Gi-protein inhibitor. Int. J. Nanomed..

[13] Tymoczko J.L., Stryer L., Stryer L., Berg J.M. (2007). Biochemistry.

[14] Rosen E.D., Spiegelman B.M. (2006). Adipocytes as regulators of energy balance and glucose homeostasis. Nature.

[15] Thompson G.R. (1989). Lipid related consequences of intestinal malabsorption. Gut.

[16] Hansen G.H., Rasmussen K., Niels-Christiansen L.L., Danielsen E.M. (2011). Dietary free fatty acids form alkaline phosphatase-enriched microdomains in the intestinal brush border membrane. Mol. Membr. Biol..

[17] van Bennekum A., Werder M., Thuahnai S.T., Han C.H., Duong P., Williams D.L., Wettstein P., Schulthess G., Phillips M.C., Hauser H. (2005). Class B scavenger receptor-mediated intestinal absorption of dietary ss-carotene and cholesterol. Biochemistry.

[18] Silverstein R.L., Febbraio M. (2009). CD36, a scavenger receptor involved in immunity, metabolism, angiogenesis, and behavior. Sci. Signal..

[19] Stahl A., Hirsch D.J., Gimeno R.E., Punreddy S., Ge P., Watson N., Patel S., Kotler M., Raimondi A., Tartaglia L.A. (1999). Identification of the major intestinal fatty acid transport protein. Mol. Cell.

[20] Buttet M., Traynard V., Tran T.T., Besnard P., Poirier H., Niot I. (2014). From fatty-acid sensing to chylomicron synthesis: Role of intestinal lipid-binding proteins. Biochimie.

[21] Iqbal J., Hussain M.M. (2009). Intestinal lipid absorption. Am. J. Physiol. Endocrinol. Metab..

[22] Mattson F.H., Volpenhein R.A. (1964). The digestion and absorption of triglycerides. J. Biol. Chem..

[23] Shen H., Howles P., Tso P. (2001). From interaction of lipidic vehicles with intestinal epithelial cell membranes to the formation and secretion of chylomicrons. Adv. Drug Deliv. Rev..

[24] Dixon J.B. (2010). Mechanisms of chylomicron uptake into lacteals. Ann. N. Y. Acad. Sci..

[25] Brunzell J.D., Hazzard W.R., Porte D.J., Bierman E.L. (1973). Evidence for a common, saturable, triglyceride removal mechanism for chylomicrons and very low density lipoproteins in man. J. Clin. Investig..

[26] Liu H., Cao X., Wang H., Zhao J., Wang X., Wang Y. (2019). Antimicrobial peptide KR-32 alleviates Escherichia coli K88-induced fatty acid malabsorption by improving expression of fatty acid transporter protein 4 (FATP4)1. J. Anim. Sci..

[27] Wang L., Llorente C., Hartmann P., Yang A.M., Chen P., Schnabl B. (2015). Methods to determine intestinal permeability and bacterial translocation during liver disease. J. Immunol. Methods.

[28] Feng S., Liu W., Zuo S., Xie T., Deng H., Zhang Q., Zhong B. (2016). Impaired function of the intestinal barrier in a novel sub-health rat model. Mol. Med. Rep..

[29] Zong X., Hu W., Song D., Li Z., Du H., Lu Z., Wang Y. (2016). Porcine lactoferrin-derived peptide LFP-20 protects intestinal barrier by maintaining tight junction complex and modulating inflammatory response. Biochem. Pharmacol..

[30] Lorente L., Martin M.M., Perez-Cejas A., Gonzalez-Rivero A.F., Lopez R.O., Ferreres J., Solé-Violán J., Labarta L., Díaz C., Palmero S. (2018). Sustained high serum caspase-3 concentrations and mortality in septic patients. Eur. J. Clin. Microbiol. Infect. Dis..

[31] Fan J.H., Feng G.G., Huang L., Tang G.D., Jiang H.X., Xu J. (2014). Naofen promotes TNF-alpha-mediated apoptosis of hepatocytes by activating caspase-3 in lipopolysaccharide-treated rats. World J. Gastroenterol..

[32] Song D., Zong X., Zhang H., Wang T., Yi H., Luan C., Wang Y. (2015). Antimicrobial peptide Cathelicidin-BF prevents intestinal barrier dysfunction in a mouse model of endotoxemia. Int. Immunopharmacol..

[33] Do-Umehara H.C., Chen C., Urich D., Zhou L., Qiu J., Jang S., Zander A., Baker M.A., Eilers M., Sporn P.H.S. (2013). Suppression of inflammation and acute lung injury by Miz1 via repression of C/EBP-delta. Nat. Immunol..

[34] Nabuurs M.J. (1998). Weaning piglets as a model for studying pathophysiology of diarrhea. Vet. Q..

[35] Shapouri-Moghaddam A., Mohammadian S., Vazini H., Taghadosi M., Esmaeili S.A., Mardani F., Seifi B., Mohammadi A., Afshari J.T., Sahebkar A. (2018). Macrophage plasticity, polarization, and function in health and disease. J. Cell. Physiol..

[36] Gao J., Wang D., Liu D., Liu M., Ge Y., Jiang M., Liu Y., Zheng D. (2015). Tumor necrosis factor-related apoptosis-inducing ligand induces the expression of proinflammatory cytokines in macrophages and re-educates tumor-associated macrophages to an antitumor phenotype. Mol. Biol. Cell.

[37] Kuo W., Lee T., Yang H., Chen C., Au Y., Lu Y., Wu L., Wei S., Ni Y., Lin B. (2015). LPS receptor subunits have antagonistic roles in epithelial apoptosis and colonic carcinogenesis. Cell Death Differ..

[38] Xiao Z., Liu L., Tao W., Pei X., Wang G., Wang M. (2018). Clostridium tyrobutyricum protect intestinal barrier function from LPS-induced apoptosis via p38/JNK signaling pathway in IPEC-J2 cells. J. Anim. Sci..

[39] Boatright K.M., Salvesen G.S. (2003). Mechanisms of caspase activation. Curr. Opin. Cell Biol..

[40] Walters J., Pop C., Scott F.L., Drag M., Swartz P., Mattos C., Salvesen G.S., Clark A.C. (2009). A constitutively active and uninhibitable caspase-3 zymogen efficiently induces apoptosis. Biochem. J..

[41] Porter A.G., Janicke R.U. (1999). Emerging roles of caspase-3 in apoptosis. Cell Death Differ..

[42] Ghoshal S., Witta J., Zhong J., de Villiers W., Eckhardt E. (2008). Chylomicrons promote intestinal absorption of lipopolysaccharides. J. Lipid Res..

[43] Plociennikowska A., Hromada-Judycka A., Borzecka K., Kwiatkowska K. (2015). Co-operation of TLR4 and raft proteins in LPS-induced pro-inflammatory signaling. Cell. Mol. Life Sci..

[44] Gaur U., Aggarwal B.B. (2003). Regulation of proliferation, survival and apoptosis by members of the TNF superfamily. Biochem. Pharmacol..

[45] Thorburn A. (2004). Death receptor-induced cell killing. Cell. Signal..

[46] Sykes M.C., Mowbray A.L., Jo H. (2007). Reversible glutathiolation of caspase-3 by glutaredoxin as a novel redox signaling mechanism in tumor necrosis factor-alpha-induced cell death. Circ. Res..

[47] Bader J.E., Enos R.T., Velazquez K.T., Carson M.S., Nagarkatti M., Nagarkatti P.S., Chatzistamou I., Davis J.M., Carson J.A., Robinson C.M. (2018). Macrophage depletion using clodronate liposomes decreases tumorigenesis and alters gut microbiota in the AOM/DSS mouse model of colon cancer. Am. J. Physiol. Gastrointest. Liver Physiol..

[48] Fredriksson L., Herpers B., Benedetti G., Matadin Q., Puigvert J.C., de Bont H., Dragovic S., Vermeulen N.P.E., Commandeur J.N.M., Danen E. (2011). Diclofenac inhibits tumor necrosis factor-alpha-induced nuclear factor-kappaB activation causing synergistic hepatocyte apoptosis. Hepatology.

[49] Kim K.Y., Stevens M.V., Akter M.H., Rusk S.E., Huang R.J., Cohen A., Noguchi A., Springer D., Bocharov A.V., Eggerman T.L. (2011). Parkin is a lipid-responsive regulator of fat uptake in mice and mutant human cells. J. Clin. Investig..

[50] Dlugosz E., Basalaj K., Zawistowska-Deniziak A. (2019). Cytokine production and signalling in human THP-1 macrophages is dependent on Toxocara canis glycans. Parasitol. Res..

[51] Sharma A.K., Fernandez L.G., Awad A.S., Kron I.L., Laubach V.E. (2007). Proinflammatory response of alveolar epithelial cells is enhanced by alveolar macrophage-produced TNF-alpha during pulmonary ischemia-reperfusion injury. Am. J. Physiol. Lung Cell. Mol. Physiol..

[52] Voortman T., van den Hooven E.H., Braun K.V., van den Broek M., Bramer W.M., Chowdhurry R., Franco O.H. (2015). Effects of polyunsaturated fatty acid intake and status during pregnancy, lactation, and early childhood on cardiometabolic health: A systematic review. Prog. Lipid Res..

[53] Neto N., Murari A., Oyama L.M., Otoch J.P., Alcantara P., Tokeshi F., Figuerêdo R.G., Alves M.J., Lima G.D.C.C., Matos-Neto E.M. (2018). Peritumoural adipose tissue pro-inflammatory cytokines are associated with tumoural growth factors in cancer cachexia patients. J. Cachexia Sarcopenia Muscle.

